# American Medical Association, Section on Stomatology

**Published:** 1901-07

**Authors:** 


### AMERICAN MEDICAL ASSOCIATION, SECTION ON STOMATOLOGY.

The American Medical Association convened at St. Paul, Minn., June 4, 5, 6, and 7, and from reports the Section on Stomatology was an unqualified success both as to numbers, quality of papers, and character of discussion. The work of this organization has always been of a high order and free from some of the objectionable features of other national bodies.

There will be presented upon the pages of this journal, until completed, full abstracts of all papers read at this meeting, together with the discussions. Several of the papers are given in the present number, together with an abstract of President Andrews's address, which forcibly outlines the true scientific and professional spirit governing this energetic branch of the great national medical organization.

